# On the Primary Dentition of Children

**Published:** 1873-03

**Authors:** Francis Minot

**Affiliations:** Cinical Lecturer on the Diseases of Women and Children in Harvard University


### SELECTED ARTICLES.

### ARTICLE IV.

### *On the Primary Dentition of Children.*

BY FRANCIS MINOT, M. D.

Cinical Lecturer on the Diseases of Women and Children in Harvard University

Gentlemen:—The subject of our lecture to-day is the Dentition of Infants, one which it is important you should be perfectly familiar with, otherwise you will be frequently embarrassed in the treatment of the diseases of a large class of those who are to come under your care. I have been struck with the fact that many young practitioners have but an imperfect acquaintance with the order in which the deciduous teeth make their appearance, and with the time of irruption of each successive group. Nor is this ignorance always confined to beginners. Physicians of many years' experience are sometimes unable to answer questions on this subject which should be familiar to every three-years' student. Not only does the condition of the primary dentition furnish an important element of prognosis in regard to the future development of the child, but any considerable departure from its normal process is apt to be attended by injurious consequences to his health. It has been well said by Vogel that although it cannot be maintained that all healthy children cut their teeth in the usual order and time, yet this much is certain, that those children who follow this order suffer the least from the difficulties and sequelæ of dentition. We will, therefore, consider now what is the normal order and time of the appearance of the temporary teeth.

You are all aware that the teeth in the human subject consist of two sets; the first of which, called the temporary or deciduous, and sometimes the *milk* teeth, twenty in number, which appear during infancy; and the second or permanent set, thirty-two in number, occupying the period between early childhood and adult life. I need hardly remind you that the germs of both sets lie hidden in the jaws of the fœtus long previous to the time of birth; but we are now only concerned with the appearance above the gum, or, as it is familiarly termed, the *cutting* of the first set—in other words, with the primary dentition.

The primary dentition begins normally between the sixth and seventh month, proceeds in a definite order, and is completed at the age of two years and a half, or a little later. Exceptions to this sometimes occur in healthy children, but they are rare, while in those of delicate constitution, or where development is retarded from various causes, there is often delay or irregularity in the evolution of the milk teeth. Hence, an acquaintance with the order of succession of these serves as a means of estimating the general state of the constitution and the degree of development of young children. Moreover, as we shall see shortly, numerous questions concerning alimentation and the diagnosis, prognosis and treatment of disease in these subjects can only be satisfactorily answered by means of this knowledge.

Authorities differ somewhat as to the order in which the first teeth appear, as well as to the time of their irruption. I have found the data given by Dr. Eichman, founded upon the observation of 400 children, to correspond pretty exactly with my own experience; and it is sanctioned by the high authority of Dr. Schœpf Merei, of Pesth, by M. Trousseau, by Vogd and others. Dr. Eichman states that the twenty deciduous teeth make their appearance in *five groups*, at five distinct periods, and in the great majority of cases in the following order:—the first group consists of the two lower median incisors, which generally appear between the 28th and the 33d week. In only three instances out of the 400 children did the first lower incisor appear as early as the 20th week, the next tooth, however, not appearing before the 31st week. Judging from my own observation, I should put six and a half months as the average date of the appearance of the first tooth. The second tooth frequently follows the first within a week, but it is not unusual for two or three weeks to elapse before the first group is completed.

The second group consists of the four upper incisors, of which the two central ones are the first to appear, the completion of the whole group requiring from three to six weeks. When a child has six teeth, therefore, there are four in the upper jaw and two in the lower. Trosseau observes that this fact, known to every woman who has had the care of young children, is strangely ignored by medical men, even by some who have written upon the very subject of dentition—and I am inclined to believe the observation is true of some practitioners of America, as well as France.

The third group includes the two lateral lower incisors and the four anterior molars. Generally, the upper molars are the first to come, next the lower incisors and lastly the lower molars.

The fourth group consists of the four canine teeth, of which the two upper are called the eye teeth, and the two lower the stomach teeth.

The fifth group comprises the four posterior molars.

Although there are exceptions to the above order, yet they are rare in healthy children. There is more variation in the age at which each group begins to appear, as well as in the length of time required for its complete evolution; but within certain limits the period is tolerably exact. The average age at which the first tooth pierces the gum may be stated at six and a half months, and the group is usually completed at seven months. The second group usually begins to appear at the age of nine months, and requires six weeks for its completion. The third group appears at the age of twelve and a half months, and is completed at fourteen months. The fourth group usually begins at eighteen months, and often requires eight weeks, and sometimes longer for its completion. The fifth group does not commonly appear before the age of twenty-six months, and also takes at least eight weeks before it is finished.

An important fact in the history of primary dentition, and which must never be lost sight of by the practitioner, is, that after the irruption of each group, there is a pause of longer or shorter duration, which is quite constant for each interval. Thus between the first and second group there is a pause of two or three months. Between the second and third group the interval is of about two months' duration. Between the third and fourth group the interval is from four to five months. Between the fourth and fifth group there is a pause of five months. I will arrange all these data in tabular form, so that you may more easily comprehend them, and retain them in memory.

1st group begins at $6\frac{1}{2}$ months—completed at 7 months.

Pause of 2 or 3 months.

2nd group begins at 9 months—completed a $10\frac{1}{2}$ months.
Pause of 2 months.
3d group begins at $12\frac{1}{2}$ months—completed at 14 months.
Pause of 4 to 5 months.
4th group begins at 18 months—completed at 21 months.
Pause of 5 months.
5th group begins at 26 months—completed at 30 months.

During the intervals between the successive groups the process of dentition is arrested, and the symptoms to which it often gives rise subside. The child is tranquil, sleeps well, is free from nervous and inflammatory troubles. Now is the time when any change which is to be made in the diet, mode of life, &c. should be begun. The important question of weaning should always be considered in reference to this period. If the mother be robust, and have plenty of milk, the child may be allowed to nurse, at least in part, with advantage until the fourth group is completed, when there will be an interval of five months in which he will be free from danger of convulsions or diarrhœa, and will have ample time to get accustomed to his new diet. Unfortunately, in a large number of cases, especially in cities, the supply of milk and the mother's strength require the child to be weaned earlier, and then, if possible, this should be done immediately after the completion of the third group, when the interval, though shorter than that which follows the canines, will yet be sufficient to permit the infant to get used to an artificial diet before the fourth begins to appear. Of course, you will not be governed by the state of the teeth alone in this important question; other considerations, especially the season of the year, the mother's health, and the supply of breast milk will have due weight; but I wish to urge upon you that in the case of delicate children, with a tendency to nervous and inflammatory symptoms, weaning can be much more safely accomplished during the intervals between the successive groups of teeth than at any other time. With perfectly healthy and robust children such precautions are not always necessary, but in delicate, town-bred infants, in those which show any disposition towards convulsions, diarrhœa, hydrocephalus, or that indefinite array of symptoms which are often styled "scrofulous," they can not be neglected without risk.

The *complications* of dentition in infants are chiefly manifested in those reflex phenomena which are due to the predominance of the spinal functions over those of the brain in early life. It is a law of the economy that in proportion as the influence of the cerebrum is suspended, or cut off, the energy of the reflex function of the spinal system is increased. Thus, during sleep, when the action of the brain is suspended, the exaltation of this function is shown by the sudden starts which may be excited by any external irritation, as tickling the soles of the feet, &c.; and the same thing may be observed in persons whose attention is completely absorbed, as while reading. So when the cerebral influence is cut off by disease or injury of the spinal cord or brain, the reflex phenomena excited by external irritation are much more energetic than in health. This you have occasionally seen exemplified in the hospital; the soles of the feet in a hemiphlegic patient being tickled, the paralyzed limb will be drawn up quickly, sometimes violently; while the other limb, over which the will still has control, may not perhaps move at all. In cases of paraplegia, this involuntary twitching of the legs sometimes arises from the accidental irritation of the feet by the contact with the bedclothes, and is then a source of much annoyance to the patients. Now the development of the brain in infancy is comparatively imperfect, and its functions are feeble, and to the predominance of the spinal over the cerebral system is due the great frequency of convulsions and other disorders at this period of life. Whether the external irritant be the pain caused by the pressure of the tooth on the gum, or the presence of indigestible food in the intestines, or worms in the rectum, an impression is sent to the cord by an afferent nerve, an impulse is carried to the muscles by an afferent nerve, and the result is those violent and irregular muscular contractions which we call spasms or convulsions.

The convulsions of dentition sometimes come on without premonitory symptoms, the child being apparently in good health at the time; but they are frequently preceded by twitchings, wakefulness, vomiting, crying and other symptoms of disturbance. A fit generally begins by twitching of the muscles of the face, which becomes pale; the eyes are at first fixed and staring, and then turned strongly to one side; the whole body becomes rigid, the limbs become violently flexed and extended, while the head is often thrown to one side, and drawn backwards. The jaws are clinched and the tongue is often bitten. The breathing is at first suspended, so that the face soon grows livid, then it becomes very rapid and noisy, a little foam (sometimes bloody, if the tongue have been bitten) issuing from between the teeth. In a large number of instances the fit lasts but a few minutes, but occasionally it is hours before the movements cease. In any case, the child remains unconscious for a considerable time after the fit is over.

In a considerable number of cases an infant never has more than one fit, but often they are repeated, and sometimes with every tooth that is cut.

The first few times you are called to a child in a fit, unless the attack be over when you reach the patient (which, fortunately, is often the case,) you will probably be puzzled to know what to do. The child will, perhaps, have been parboiled by being plunged in a hot bath; he has been anointed with goose grease, and poulticed with onions, *usque ad nauseam;* ipecac has been forced down his throat, and soapsuds into his rectum. The family is in consternation, and in their eagerness to tell you what they have done (which is the thing uppermost in the minds of a great many patients when they consult a doctor) you will find it difficult to get any account of the symptoms of the case. Recollect that in most cases there is very little to be done. The attack will generally come to a favorable end without any interference. In case it should show no signs of abatement, the best remedy is ether by inhalation. As soon as the patient comes under its influence, the spasmodic action of the muscles cease, and he will soon fall into a quiet sleep, from which he should not be roused for hours. At your second visit, the gums should be carefully examined, and an incision should be made across any one which is swollen and hard, and such general directions as to the diet and hygienic treatment may be given as the case seems to require. Your opinion may be asked as to the danger of a recurrence of the convulsions. In answering this question you will be guided by the general state of the patient's health, and partly by the progress which the dentition has made. If the child be feeble and delicate, with a large anterior fontanelle, at the age of twelve months, there is reason to fear an irritability of the nervous system, which will find expression in convulsions when there is much peripheral irritation. So if the fourth group of teeth are just beginning to appear, there is danger that the attack will recur, perhaps more than once, before all the canines are through the gum. If, on the other hand, the child be well developed and healthy look, if the hygeinic surroundings are good, if one group of teeth is nearly completed, then there is reason to hope that the convulsions may not recur.

When a child seems predisposed to convulsive attacks, or if it have had more than one attack, the bromine of potassium will often be found of great service as a prophylactic. This medicine owes its value in these and similar affections to its remarkable sedative power over the reflex function of the spinal marrow. From five to fifteen grains, according to the age of the child, may be given at a time, when there is threatening of convulsions, and this dose may be repeated twice or thrice daily, according to circumstances. In many cases it will be found to be perfectly efficacious. Formerly assafœtida, in enema, was given for this purpose, but the bromine is much more certain in its effects, besides far less disagreeable. Many children who are subject to convulsions during teething are pale, ill-nourished and feeble. The best prophylactics for children are cod-liver oil and some preparation of iron. Of the former, from half a teaspoonful to a teaspoonful may be given three times daily. With regard to iron there is no choice among the large number of preparations except so far as facility of administration goes. I usually prescribe the tartrate of iron and potassa for children, because it is less disagreeable to the taste and less constipating than most others. From two to five grains may be given, three times daily, dissolved in sweetened water.

The diarrhœa, which sometimes complicates teething, is chiefly of importance during hot weather. It varies in amount from a little looseness of the bowels to a large number of discharges daily. In any case, you should watch it carefully, and endeavor to restrain it if it become at all urgent. Formerly a little diarrhœa was thought to be rather beneficial than otherwise during the cutting of a tooth, but I believe this opinion is no longer held. At any rate, I advise you to give it no quarter in hot weather, as it then too often ends in cholera infantum, a disease which destroys from 50 to 80, or more, children every week in this city, in the summer months. On the first appearance of any looseness of the bowels the teeth should be carefully looked to, and the gums lanced if necessary. If there be evidence of indigestible food in the intestine, a laxative of castor oil or rhubarb may be given. After this has acted, the remedies to be employed are astringents and sometimes opiates. The common chalk mixture answers very well in many cases, in the dose of one or two teaspoonfuls, repeated as often as required. I am in the habit of adding to this one-part of tincture of kino, which increases its astringency, and also keeps it from turning sour in hot weather. The dose is the same. If the diarrhœa be not checked by this mixture, one drop of laudanum may be added to a dose of it, but not oftener than three times a day, in children under 2 years old. Diarrhœa is most apt to attack children who are brought up on the bottle; hence, if the case be urgent and do not yield to treatment, a wet nurse should be procured if possible. When this cannot be done, I would strongly recommend the method of preparing milk with arrow-root and gelatine, which is to be found in the Diseases of Children, by Drs. Meigs and Pepper, and which Dr. Charles P. Putnam speaks so favorably of in his valuable and interesting article on *Artificial Food for Young Infants*, which was printed in the *Boston Medical and Surgical Journal* for August 1, 1872. Recollect that these young subjects run down rapidly, and that the treatment and diet must both be of a sustaining character. There is nothing which seems to be so useful to a teething child, exhausted by diarrhœa, as brandy, which should be given once in three or four hours, or oftener in urgent cases. The dose is from five to twenty-five drops ordinarily—but if there be much prostration you need not fear to increase the amount. It is best given in milk.

Wine-whey suits many children admirably when they are suffering from diarrhœa. It should be diluted about one-half for infants, and may be given in large quantities instead of the ordinary diet. You will often be asked how to make it; the proportions are, a wineglassful of sherry wine to a tumblerful of milk; heat the milk, and just as it begins to boil pour in the wine. In a few minutes the curd separates and the whey may be strained off. It should be sweetened.

You must not suppose that either convulsions or diarrhœa are inevitable during dentition. With healthy children who are nursed they are exceptional. But it is rarely that even healthy children escape without some indisposition while cutting their teeth. In most cases this amounts only to fretfulness, loss of appetite, slight delirium, &c. Occasionally I have seen a child lie in a state approaching to stupor for several days, without any other symptom, its condition being evidentally due to the process of dentition. There are cases of genuine, though slight, traumatic fever. They usually require no treatment beyond shelter from noise and light, a somewhat restricted diet, and bromine of potass. or small doses of Dover's (one to two grains) at night, with plenty of water to drink.—*Boston Medical and Surgical Journal.*

---

